# A Real-World Study on Ge Gen Tang in Combination with Herbal Medicines for Relieving Common Cold-Associated Symptoms

**DOI:** 10.1155/2022/4790910

**Published:** 2022-07-22

**Authors:** Pei-Ying Chou, Chen-Jei Tai, You-Jen Tang, Yu-Chuan Chen, Kung-Yi Lin, Ching-Chiung Wang

**Affiliations:** ^1^Ph.D. Program in Clinical Drug Development of Herbal Medicine, Taipei Medical University, Taipei, Taiwan; ^2^Department of Pharmacy, Taipei Medical University Hospital, Taipei, Taiwan; ^3^Department of Obstetrics and Gynecology, School of Medicine, College of Medicine, Taipei Medical University, Taipei, Taiwan; ^4^Graduate Institute of Pharmacognosy, College of Pharmacy, Taipei Medical University, Taipei, Taiwan; ^5^Department of Traditional Chinese Medicine, Chiayi Branch, Taichung Veterans General Hospital, Chiayi, Taiwan; ^6^Consultant, Taipei, Taiwan; ^7^School of Pharmacy, College of Pharmacy, Taipei Medical University, Taipei, Taiwan; ^8^Traditional Herbal Medicine Research Center, Taipei Medical University Hospital, Taipei, Taiwan

## Abstract

**Purpose:**

Real-world evidence refers to patient data derived from the healthcare process. In this study, we used National Health Insurance Research Database (NHIRD) assessments and clinical studies of Ge Gen Tang (GGT, 葛根湯) in patients with common cold to establish a real-world study model of Traditional Chinese Medicine formulae. GGT is widely prescribed for the treatment of common cold in Taiwan, generally in combination with other medicines. The aim of this study was to determine whether a correlation exists between GGT combined with other medicines and an improvement in cold symptoms. We also established a GGT prescription compatibility system by analyzing Taiwan's NHIRD records for GGT prescription patterns in patients with different types of common cold.

**Materials and Methods:**

We extracted and analyzed records from the NHIRD for the period 2000–2015 to determine the most common clinical applications of GGT. GGT and GGT with Chuan Xiung Cha Tiao San were most commonly prescribed for common cold, as per NHIRD recommendations. Records for adults aged 20–65 years who were prescribed GGT for the treatment of common cold (Diagnosis Code ICD-9-460) were included in this study. We assessed the following indicators of the common cold, before and after treatment with GGT: nasal congestion, cough, runny nose, sneezing, sore throat, hoarseness, stiff shoulder, headache, and general physical condition.

**Results:**

The cold symptom scores before and after taking the GGT prescriptions significantly differed in the 29 volunteers. The 29 volunteers reported a significantly lower headache severity score after medication than before medication (*p* < 0.004). Furthermore, patient scores for general physical condition decreased significantly (*p* < 0.01) after medication.

## 1. Introduction

Real-world data (RWD) is the basis of real-world evidence (RWE) and can be collected from electronic health records, claims and billing activities, and patient-generated data, including in home-use settings [1, 2]. RWE is the conclusion of the proper statistical analysis of RWD that includes drafting an appropriate research design and statistical analysis methods, collecting appropriate RWD according to the research design, and performing data analysis so that analysis results that meet the research objectives can be generated. RWE can provide information about clinical settings and the health-system characteristics that influence treatment effects and outcomes [3]. This real-world study design included an analysis of National Health Insurance Research Database (NHIRD) records and clinical studies of Ge Gen Tang (GGT) in the common cold.

GGT was recorded as an herbal formulation in the ancient text of Chinese medicine, Shang Han Lun (傷寒論). GGT formulations typically contain Puerariae radix, Cinnamomi Ramulus, Paeoniae Radix, Ephedrae Herba, Glycyrrhizae Radix, Zingiberis Rhizoma, and Jujubae Fructus. In traditional Chinese medicine (TCM) theory, wind (風邪), which is one of the six excesses (六邪), is the main cause of various types of common cold. Wind-cold (風寒) syndrome usually occurs in late autumn and winter, whereas wind-heat (風熱) syndrome usually occurs in summer. The incidence of the common cold is greater during spring and winter, with wind-cold and wind-heat syndromes being the most common. Wind-cold syndrome is characterized by an aversion to cold along with a fever without any sweating, a runny nose, scratchy throat, and cough. Wind-heat syndrome is characterized by fever, thick nasal discharge, cough, and yellowish purulent sputum [4]. According to TCM theory, indications for prescribing GGT include symptoms of wind-cold syndrome (i.e., headache, fever, aversion to cold and no sweating, and contracture of the nape and neck). A traditional Chinese medical physician follows eight principles (ba gang, 八綱) as a guideline for the diagnosis of these syndromes. These eight principles are: Yin (陰), Yang (陽), Exterior (表), Interior (裏), Cold (寒), Heat (熱), Deficiency (虛), and Excess (實). According to Ba Gang, GGT contains seven ingredients with different properties for different treatments. These ingredients include Puerariae radix for exterior-heat-deficiency, Ephedrae Herba for exterior-cold-excess, Cinnamomi Ramulus for exterior-cold-deficiency, Glycyrrhizae Radix for exterior-deficiency, Paeoniae Radix Alba for heat-deficiency, and Zingiberis Rhizoma and Jujubae Fructus for cold-deficiency. Therefore, via GGT, the combination of the above seven ingredients, can be used to treat exterior cold (heat) deficiency (excess) symptoms. The exterior-cold-excess syndrome arises when wind-cold invades the exterior and is characterized by a pronounced aversion to cold and mild fever, headache, generalized pain, absence of sweating, thin white tongue coating, and tight floating pulse, showing that the external part of the body is being attacked by cold, although the patient's defense qi is not damaged. However, Gui Zhi Tang can be used for exterior cold-deficiency syndrome (the patient's defense qi is damaged, manifested by intolerance of wind, persistent sweating, fever, headache). GGT is Gui Zhi Tang plus Puerariae radix and Ephedrae Herba and is used for “tonic convulsion.” (exterior syndrome due to wind-cold marked by rigidity of the neck without sweating). Therefore, GGT is used to treat exterior-cold-deficiency syndrome to a greater extent than Gui Zhi Tang. Its state belongs to the exterior-cold-excess syndrome but is lower than the state of Ma Huang Tang [5]. In clinical practice, TCM physicians may comprehensively consider whether to prescribe GGT according to the symptoms of the common cold using the Ba Gang syndrome differentiation, in addition to other methods. In this study, we used clinical observations of GGT prescription patterns in patients to assess the GGT guidelines for treating the common cold.

Previous research has demonstrated that GGT is effective against human respiratory syncytial virus-induced plaque formation and stimulates mucosal cells to secrete interferon beta to counteract viral infection [6]. One experimental dog model revealed that body temperature and macrophage phagocytic activity increased 30 min after administering GGT [7]. Furthermore, a clinical trial proved that the beneficial effect of GGT on shoulder stiffness was due to an improvement in blood circulation, resulting in higher body surface temperature [8]. A study of the data obtained from the NHIRD between 2005 and 2007 revealed that GGT was the most commonly used formula for acute nasopharyngitis, chronic nasopharyngitis, and allergic rhinitis [9].

This study analyzed NHIRD records to determine GGT prescription patterns for patients with different types of common cold, as defined by TCM, and with cold symptoms, as described by Western medicine. The correlation between treatments using GGT combined with various other medicines and improved cold symptoms was used to establish a GGT prescription compatibility system for the common cold. Clinically, GGT is widely prescribed in Taiwan for the common cold, generally in combination with other medicines. However, the prescription patterns for GGT combined with other medicines have not previously been explored. Therefore, we used a real-world study design to investigate the clinical efficacy of GGT in patients with common cold and compared our findings with NHIRD to establish the clinical use guidelines of GGT for treatment of the common cold.

## 2. Materials and Methods

### 2.1. Prescription Patterns of GGT in NHIRD

The current study analyzed the records of one million individuals from the total population that were available on NHIRD between 2000 and 2015. We documented instances when GGT was prescribed, and the prescription patterns of GGT alone or in combination with other medicines were collated with reference to the diagnosis codes. We screened for diseases with the most frequently occurring prescription patterns, including GGT. Then, the contents of the prescriptions for common cold were analyzed to determine the most common prescription contents.

### 2.2. A Clinical Observation Study

A study was conducted with enrolled patients who visited the Department of Traditional Chinese Medicine at Taipei Medical University Hospital. The protocol was registered with the Taipei Medical University-Joint Institutional Review Board (Approval No. N201509008). In this study, GGT was prescribed in the form of concentrated medicine granules. The ratio between each herb of GGT is per 10 g contains Puerariae radix 6.0 g, Cinnamomi Ramulus 3.0 g, Paeoniae Radix 3.0 g, Ephedrae Herba 4.5 g, Glycyrrhizae Radix 3.0 g, Zingiberis Rhizoma 4.5 g, and Jujubae Fructus 4.0 g.

Individuals aged 20 to 65 years who had been prescribed GGT for the treatment of the common cold (Diagnosis Code ICD-9-460) were enrolled in the study. Patients who were pregnant, consumed other Western medicines or herbal products along with GGT for the common cold, or did not maintain a healthy diet during the observation period were excluded.

**Figure 1:**
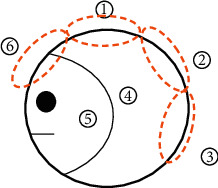
Headache locations.

After seven days of taking their prescribed medication, the participants revisited their physicians. The participants were asked to complete a daily questionnaire that assessed the nature of their cold symptoms, including the location and severity of any headache, their body temperature, and their general physical condition. Their headache location was described as follows: 1. top of the head, 2. back of the head, 3. rear neck, 4. sides of the head, 5. near the temples, and 6. front of the head (Figure 1). The patients were asked to assess their symptoms based on those determined in a previous randomized controlled trial [10]. The items included 13 specific complaints: runny nose, nasal congestion, frequent need to blow their nose, thick mucus discharge, sneezing, sore throat, scratchy throat, cough, chills, hoarseness, stiff shoulder, joint pain, and general malaise. The patients were asked to score their symptoms daily, based on five grades, where grade 0 was defined as no symptoms of the common cold; grade 1 as a few instances of the symptoms on that day; grade 2 as frequent occurrences of symptoms that did not cause difficulties in daily life; grade 3 as frequent occurrences of symptoms that caused difficulties in daily life; and grade 4 as the occurrence of severe symptoms of the common cold on that day. General physical conditions were classified using scores of 0 to 10, where a score of 10 indicated that the patient's general physical condition was seriously impacted. Participants kept a record of their headache severity score four times every day and reported their daily value for this symptom as the mean of their four headache severity readings. Any improvement in the severity of the cold symptoms was correlated with the patients' GGT prescription pattern. We also reviewed the patients' medical records for the following information: prescriptions, main symptoms of the participants, and their tongue, pulse, and TCM syndrome types, as confirmed by the TCM physician.

## 3. Study Analysis

Statistical analyses were performed using SPSS 18 software. Patient scores for cold symptoms, general physical condition, and headache severity between “before medication.” and “on the fifth day after medication.” were compared using a paired samples *t*-test. Body temperature readings between before medication and on the fifth day after medication were compared using a one sample *t*-test. The results of the *t*-tests were presented as the mean ± standard deviation, and *p* < 0.05 was considered statistically significant.

## 4. Results

### 4.1. Prescription Patterns of GGT for the Common Cold in NHIRD

We found the most common disease for which GGT was prescribed was acute nasopharyngitis (common cold, diagnosis code ICD-9-460), and approximately 20.18% of a total of 1,856,166 GGT prescriptions were for acute nasopharyngitis (common cold, ICD-9 460). The second most frequent indication was symptoms involving the head and neck (ICD-9 784) (Table 1). An analysis of the herbal components most commonly used in combination with GGT was performed among the 374,499 prescriptions. The most frequent herbal combination components for the common cold could be divided into five patterns: GGT alone; GGT combined with Chuan Xiung Cha Tiao San (CXCTS); GGT combined with Yin Qiao San (YQS); GGT combined with Xin Yi San (XYS); and GGT combined with Chiu Wei Chiang Huo Tang (CWCHT) (Table 2). GGT alone and GGT combined with CXCTS were the most common prescriptions for the common cold in the data obtained from the NHIRD.

### 4.2. A Retrospective Clinical Observation Study

Of the 30 patients enrolled in the study, 29 were included in the analysis, while one who consumed other medicine in this period was excluded. All body temperature readings in all participants during the study were below 37.5°C (Table 3). Most of the participants were women.

Table 4 presents data relating to the participants and their TCM-defined syndromes. Eight participants were diagnosed with wind-cold-exterior syndrome (excess-syndrome), and 14 were diagnosed with deficiency syndrome and reported muscle soreness, night cough, chills, runny nose, dizziness, white phlegm, and neck stiffness. Participants diagnosed with wind-cold-exterior dampness syndrome had symptoms of wind-cold-exterior syndrome, as well as an index finger with prurigo, heavy-headedness, headache, and painful stiff neck. Participants with wind-heat-exterior syndrome had throat suppuration.

The TCM common cold syndrome definitions as related to different symptoms are presented in Table 5. Differences in the patients' scores for their general physical condition before medication and on the fifth day after medication were significant (Table 6). This implies that the general physical condition of the patients improved after medication. A total of 16 participants reported headaches. Compared to their scores before medication, the headache frequencies reported for each location (refer to Figure 1) on the fifth day after medication were as follows: headache frequencies at locations 1, 3, 4, 5, and 6 decreased. The severity of headache at location 4 (sides of the head) decreased significantly, indicating quick recovery. At location 2 (back of the head), headache severity was higher on the fifth day after medication. However, a more detailed review of the headache severity records revealed that the location of the headache before and after medication often differed. For example, the reporting of headaches at location 2 was less frequent before medication than on the fifth day after medication. By contrast, the frequency of reporting a headache at location 6 was higher before medication and lower on the fifth day after medication, indicating that the headache location changed during the course of the common cold (Table 7). A significant difference in headache severity was noted between before medication and on the fifth day after medication, according to the results of the paired *t*-test (Table 6).

Patient surveys for each cold symptom, including runny nose, nasal congestion, cough, sneezing, sore throat, hoarseness, stiff shoulder, chills, and general malaise, indicated significant differences between the scores given before medication and on the third day after medication (*p* value in column A of Table 8). Significant differences were also observed for scores before medication and those on the fifth day after medication (*p* value in column B of Table 8). However, no significant differences in nasal congestion and stiff shoulder conditions were observed between scores reported before medication and on the third day (*p* values in column A of Table 8). This indicates that except for nasal congestion and stiff shoulders, cold symptoms were alleviated on the third day after medication. This study included a review of chart reports for patients with common cold that documented patient age and prescriptions. We found that there were 20 different combinational prescriptions for GGT and 35 single-herb prescriptions. Based on their symptoms, patients were divided into wind-cold syndrome or wind-dampness syndrome groups, and GGT prescription patterns were compared. The GGT prescription that was used to treat the common cold comprised a single dose between 2.0 and 3.3 g, which was repeated three times a day, to total a daily GGT dose between 6.0 and 10.0 g.

Ma Huang Fu Zi Xi Xin Tang (MHFZXXT) was used to treat headaches in two patients. Qiang Huo Sheng Shi Tang (QHSST) was used to treat headaches in patients with wind-cold-dampness syndrome. Furthermore, MHFZXXT and QHSST synergized with GGT to treat headache and neck stiffness, respectively. Qing Bi Tang (QBT) alleviated the thick mucosal discharge symptoms for one patient, while Jin Fey Tsao Saan (JFTS) alleviated the cough symptoms of two patients. Jie Geng (*Platycodon grandiflorum*), as a single herb prescription, improved sores and alleviated scratchy throat symptoms in four patients. Kan Chiang (*Zingiber officinale*) and Xin Gren (*Prunus armeniaca*) were used to treat coughs in five and two patients, respectively. Kan Chiang with Xin Gren ameliorated the cough symptoms of one patient.  Table 9 displays the list of physician-prescribed single herbs and formulae that were reported to alleviate cold symptoms. The patients' scores for single herb and formulae prescriptions were compared before and after medication. Symptoms were deemed to have improved if the scores decreased by one or more points.

## 5. Discussion

The NHIRD contains RWD from physician clinical medication records. Table 2 shows that GGT alone and in combination with CXCTS were the most common prescriptions for the common cold. The effect of CXCTS is to dispel wind and relieve pain. In a study on TCM prescription patterns for patients with migraines in Taiwan, CXCTS was found to be the most frequently prescribed treatment [11]. GGT is mainly used for exterior cold-deficiency syndrome, which when combined with CXCTS, YQS, XYS, or CWCHT, is not limited to treating solely deficiency syndrome. Moreover, the compatibility of GGT and YQS will not be limited for only cold or heat syndromes of the common cold.

The research design of this study was based on a real-world study. The TCM physicians issued prescriptions based on the patient's cold symptoms, and we did not provide any limitations. When the patient took a prescription with GGT, we used a questionnaire to evaluate the improvement of his or her symptoms to analyze the prescription patterns for the common cold.

We discovered five types of GGT prescription patterns for the common cold that corresponded to TCM definitions of disease location (exterior-interior), strength (deficiency-excess), and symptoms (cold-heat). All the GGT prescription patterns that were documented in our dataset were for exterior-cold-excess, with the exception of YQS for exterior-heat-excess syndromes. According to TCM theories, we divided GGT prescriptions into five types for different common cold symptoms (Table 2).

We concluded that exterior (表) was the most common TCM diagnostic condition that led to GGT prescriptions for the common cold. Symptoms such as runny nose, nasal congestion, a need to frequently blow the nose, and thick mucosal discharge always occurred together in our dataset. A significant improvement was observed in patient scores for these nasal congestion symptoms by the fifth day after medication.

In Western medicine, antihistamine medication is often prescribed for a runny nose, and a bronchodilator, such as dextromethorphan, is used for a cough or asthma. We believe this is equivalent to TCM prescriptions for GGT combined with QBT for a thick mucosal discharge and GGT combined with Xin Gren for a cough. Notably, there was an advantage of no daytime sleepiness being reported when GGT was combined with herbal medicine for these symptoms.

During this study, the body temperature of all participants was consistently lower than 37.5°C. The most frequently reported symptoms were a cough, the need to frequently blow the nose, and a scratchy throat. The patients who received GGT alone experienced some relief from these reported cold symptoms, and those who received GGT combined with other formulae or herbs reported improvements in other symptoms. For example, GGT combined with MHFZXXT was reported to relieve headaches, and GGT combined with Jie Geng was reported to improve sore throats.

Overall, GGT was often used to treat common cold symptoms, both alone and in combination with other prescriptions, including MHFZXXT, QHSST, QBT, JFTS, Jie Geng, Kan Chiang, and Xin Gren. Based on TCM theory, the principle of medicine formulation follows a “sovereign, minister, assistant, and courier.” theory. This clinical study revealed that prescription patterns for the common cold were based on “sovereign, minister, assistant, and courier.” theory principles. GGT was mainly used under the sovereign theory with doses that ranged from 3.0 to 3.3 g (three times a day, TID).

In TCM theory, the head is the confluence of all yang meridians. The common cold attacks the greater yang meridian, causing a headache. Headache locations corresponded to yang meridians as follows: location 3, the greater yang meridian (太陽經); location 4, the gallbladder meridian (膽經); and location 6, the yang brightness meridian (陽明經). Patients commonly reported a headache in locations 3, 4, and 6 before medication. After medication, patient headache scores in these three locations decreased, whereas the headache score at location 2 was often higher after medication than before. This implies that GGT in combination with other formulae can alleviate headaches of the greater yang meridian, gallbladder meridian, and yang brightness meridian.

We found that headaches associated with the common cold were associated with external wind and cold. The 16 participants who reported a headache were prescribed a combination formula of MHFZXXT and QHSST based on the TCM diagnosis and treatment related to the wind-cold-dispersing medication. MHFZXXT has been documented in the Shang Han Lun as a substance that warms the meridian, dissipates cold, reduces blood histamine content, and promotes nasal mucosa recovery [12] (Table 10). In TCM, the common cold is described as having wind-cold and wind-heat syndromes, and TCM physicians prescribe medicines based on the differences between these syndromes. According to the questionnaires that the participants completed (Table 8), GGT combined with other formulae and herbs relieved common cold-associated symptoms (Table 9). A prior observational study that compared visual analog scales before and after the application of a GGT combined with MHFZXXT medication for 100 patients with migraines observed a significant improvement in symptoms [19]. Notably, MHFZXXT was used for exterior-effusing, warming the meridian, and dissipating cold (Table 10), whereas GGT was used to treat cold wind headache. According to Table 10, we speculate that MHFZXXT can relieve a wind-cold headache. The combination dose of GGT with MHFZXXT ranged from 0.9 to 1.0 g (TID).

QHSST belongs to the TCM category of pungent and warm formulae and promotes sweating and dispelling of wind and dampness syndrome symptoms. In a randomized controlled study, the combination of GGT and QHSST was compared with acupuncture for the treatment of cervical spondylosis. The results revealed that 96.30% of 54 patients who received GGT combined with QHSST exhibited improvements [13]. In this study, QHSST was administered in combination with GGT for the wind-dampness headache, and the dose ranged from 1.2 to 2.0 g (TID).

QBT is a wind-cold-dispersing medicine that can clear heat and relieve a stuffy nose and chronic sinusitis [14]. In a clinical observation study, QBT treatment improved nasal congestion symptoms in 68 patients. The QBT ingredients include Platycodonis Radix, which promotes qi circulation and removes dampness, and Scutellariae Radix, which clears heat and expels pus [20]. The dosage of QBT that was administered was 1.0 g (TID). GGT was combined with QBT to relieve nasal congestion and stuffy noses.

JFTS also belongs to the TCM pungent and warm formulae category and can direct qi downward to resolve phlegm [15]. In an observational study, JFTS was compared with Western medicine for the treatment of cough symptoms in patients with wind-cold syndrome. After five days, cough symptoms improved in patients who received JFTS [21]. Additionally, Jie Geng contains Platycodonis Radix, which diffuses in the lungs and eliminates phlegm, promotes throat comfort, expels pus, and inhibits expression of nuclear factor-*κ*B, caspase-3, and Bax [16]. Kan Chiang was found to warm the center, disperse cold, return yang, and reduce the expression of IL-17 and IL-23 in experimental mice with autoimmune encephalomyelitis [17]. To summarize, in prescriptions that improve symptoms of the common cold, GGT with QHSST was used for headaches; QHSST was used for wind-dampness headaches assailing the exterior; and MHFZXXT was used for headaches associated with cold wind syndrome with yang deficiency, exterior cold pattern, and long-term clinical effects for allergic rhinitis. QBT was used for treating nasal mucus in cold wind syndrome and interior heat patterns. JFTS was used for treating a cough and white sputum in cold wind syndrome. Xin Gren and Jie Geng were used for cough and throat pain, respectively, in cold wind and hot wind syndromes.

## 6. Conclusion

In NHIRD research, GGT alone and combined with CXCTS were the most common prescriptions for the common cold. We explored GGT and found the formula to be effective for treating runny nose, nasal congestion, sneezing, chills, stiff shoulder, joint pain, and general malaise of wind-cold syndrome. We also noted a statistically significant difference between cold symptoms before and after medication. Notably, the differences in patient headache and general physical condition scores before and after medication were statistically significant.

**Figure 2:**
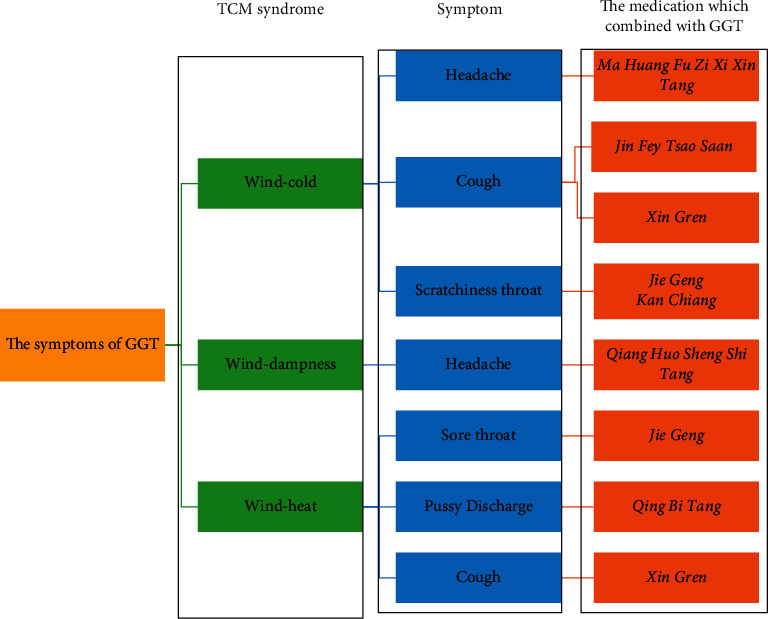
Applications of GGT and its prescription compatibility for the common cold. (a) TCM syndrome. (b) Symptom. (c) The medication which combined with GGT.

Figure 2 presents a summary of the TCM syndromes of wind, wind-cold, wind-heat, and wind dampness and the common prescriptions used to treat each condition. A combination of GGT and MHFZXXT was commonly used for headaches due to wind-cold syndrome with yang deficiency and exterior cold patterns. GGT with JFTS was often used for cough due to wind-cold syndrome with white sputum.

## Figures and Tables

**Table 1:** Top 10 diseases for which treatment prescriptions contain GGT (according to the frequency distribution of ICD-9-CM code).

No.	Code	Disease	%
1	460	Acute nasopharyngitis (common cold)	20.18
2	784	Symptoms involving head and neck	11.95
3	477	Allergic rhinitis	8.3
4	729	Other disorders of soft tissues	4.91
5	472	Chronic pharyngitis and nasopharyngitis	4.55
6	786	Symptoms involving respiratory system and other chest symptoms	4.17
7	780	General symptoms	3.42
8	723	Other disorders of cervical region	3.26
9	724	Other and unspecified disorders of back	3.14
10	487	Influenza	2.11

**Table 2:** The frequency and indications for the application of GGT prescriptions^a^ for the common cold.

Type	Formula	Frequency (%)	Applied syndrome	External pathogen	Indication
1	GGT	1.64	Exterior-cold-excess	Wind-cold	Headache, fever, aversion to cold, contracture of the nape and neck

2	GGT CXCTS	0.81	Exterior-cold-excess	Wind-cold	Headache, migraine, fever, aversion to cold, contracture of the nape and neck

3	GGT YQS	0.55	Exterior-cold-excess Exterior-heat-excess	Wind-cold	Headache, fever, thirst, sore throat

4	GGT XYS	0.55	Exterior-cold-excess	Wind-cold	Headache, fever, runny nose, nasal congestion

5	GGT CWCHT	0.51	Exterior-cold-excess	Wind-cold-dampness	Headache, fever, stiffness of the neck, postnasal drip

'a' represents total number of prescriptions, *n* = 374,499.

**Table 3:** Characteristics of the participants who received GGT prescriptions for common cold treatment (ICD-9-460).

Characteristic		No
Sex	Male	8
	Female	21

Age (years)	21–30	8
	31–40	6
	41–50	7
	51–65	8

Body temperature	<37.5°C	29
	>37.5°C	0

Taking other medication during the treatment period	Yes	1
	No	29

**Table 4:** Objective and subjective symptoms of the common cold based on the classification of TCM syndrome.

TCM syndrome	Identified by TCM physician		No	Subjective symptom
	Numbers of excess syndrome	Numbers of deficiency syndrome		
Wind-cold-exterior	8	14	22	Muscle soreness, cough at night, chills, runny nose, dizzy, white phlegm, and neck stiffness

Wind-cold-dampness-exterior	2	4	6	Index finger with prurigo heavy-headedness and headache and painful stiff nape

Wind-heat-exterior	1	0	1	Throat suppuration

**Table 5:** TCM syndrome of the common cold based on different symptoms.

TCM syndrome	Symptom of common cold
Wind-cold syndrome	Scratchy throat
	Sore throat (no thirst)
	Cough (cold-phlegm)

Wind-heat syndrome	Sore throat (thirst)

Wind-heat syndrome	Pussy discharge
Wind-dampness syndrome	Cough and yellowish purulent sputum

Wind-cold syndrome	Headache
Wind-heat syndrome	
Wind-dampness syndrome	

**Table 6:** Comparison of headache severity and general physical condition before and after medication.

Variable	No	Before medication	Fifth day after medication	*p* value
Headache severity	16	4.38 ± 3.519	1.63 ± 1.784	0.004^*∗∗*^
General physical condition	27	5.41 ± 2.308	3.19 ± 2.001	0.001^*∗∗∗*^

^*∗*^ *p* < 0.05,^*∗∗*^*p* < 0.01,^*∗∗∗*^*p* < 0.001.

**Table 7:** Distribution of headache locations before and after medication.

Location	Before medication (%)	After medication				
		Day 1(%)	Day 2 (%)	Day 3 (%)	Day 4 (%)	Day 5 (%)
1	1.56	1.30	1.56	0.78	0.52	0.52
2	0.00	0.26	0.00	1.04	1.56	2.08
3	2.60	3.13	2.34	2.60	2.60	1.04
4	2.60	0.00	0.52	0.78	0.00	0.52
5	1.82	1.82	1.30	1.56	0.78	1.04
6	2.60	2.60	2.34	1.56	2.34	0.52

*N* = 384.

**Table 8:** Comparison of daily cold symptoms before and after medication.

Cold symptom	No	Before	Day 3	Day 5	*p* value 1	*p* value 2
Runny nose	14	2.07 (0.92)	0.85 (0.77)	0.64 (0.63)	0.001^*∗∗∗*^	0.001^*∗∗∗*^
Nasal congestion	15	1.60 (0.98)	1.13 (0.91)	0.67 (0.72)	0.131	0.002^*∗∗*^
Blow nose	17	1.65 (0.93)	0.88 (0.78)	0.70 (0.68)	0.005^*∗∗*^	0.0001^*∗∗∗*^
Pussy discharge	12	1.66 (0.98)	0.91 (0.67)	0.4 (0.51)	0.043^*∗*^	0.004^*∗∗*^
Sneezing	14	1.57 (0.94)	0.78 (0.57)	0.35 (0.49)	0.01^*∗∗*^	0.001^*∗∗∗*^
Sore throat	15	2.33 (1.17)	0.64 (0.84)	0.33 (0.61)	0.001^*∗∗∗*^	0.0001^*∗∗∗*^
Scratchy throat	16	2.31 (1.3)	1.12 (1.08)	0.56 (0.81)	0.005^*∗*^	0.0001^*∗∗∗*^
Cough	20	2.00 (1.07)	1.35 (1.08)	0.95 (0.94)	0.044^*∗*^	0.001^*∗∗∗*^
Chills	9	1.56 (0.72)	0.6 (0.69)	0.56 (0.72)	0.019^*∗*^	0.04^*∗*^
Hoarseness	14	2.00 (0.78)	1.00 (1.17)	0.50 (0.76)	0.020^*∗*^	0.0001^*∗∗∗*^
Stiff shoulder	13	1.92 (0.76)	1.54 (0.31)	1.38 (0.76)	0.096	0.003^*∗∗*^
Joint pain	5	1.8 (0.83)	0.6 (0.89)	0.6 (0.89)	0.033^*∗*^	0.033^*∗*^
General malaise	15	1.8 (1.10)	0.86 (1.18)	0.46 (0.63)	0.001^*∗∗∗*^	0.001^*∗∗∗*^

*p* value 1 is the value obtained using the paired *t*-test, comparing daily cold symptoms before and 3 days after medication. *p* value 2 is the value obtained using the paired *t*-test, comparing daily cold symptoms before and 5 days after medication.

**Table 9:** List of physician-prescribed reused single herbs and formula that improve cold symptoms.

Cold symptoms	Single herb			Formula			
	Xin Gren	Kan Chiang	Jie Geng	Qing Bi Tang	Ma Huang Fu Zi Xi Xin Tang	Qiang Huo Sheng Shi Tang	Jin Fey Tsao Saan
Dosage (TID)	0.3 g	0.3 g	0.3 g	1.0 g	0.9–1.0 g	1.2–2.0 g	1.0–2.0 g
Headache	●	●	●		●	●	
Stuffy nose	●	●	●	●	●		
Sore throat	●	●	●				
Scratchy throat	●	●	●			●	
Cough	●	●	●	●		●	●

**Table 10:** List of reused single herbs and formula and their functions and pharmacological effects on cold symptoms in patients.

Main prescription	Combination prescription	Combination/origin	Function of combination prescription	Pharmacological effects of combination prescription
Ge Gen Tang	Ma Huang Fu Zi Xi Xin Tang	Ephedrae Herba, Aconiti Lateralis Radix Praeparata, and Asari Radix et Rhizoma	Exterior-effusing and dissipate cold or warm the meridian and dissipate cold	Antiallergy: Ma Huang Fu Zi Xi Xin Tang reduces blood histamine content and recovers the nasal mucosa [12]
	Qiang Huo sheng Shi Tang	Notopterygii Rhizoma et Radix, Angelicae Pubescentis Radix, Ligustici Rhizoma et Radix, Saposhnikoviae Radix, Glycyrrhizae Radix et Rhizoma Praeparata cum Melle, Viticis Fructus, and Chuanxiong Rhizoma	Promotes sweating, dispels wind, and dispels dampness	Gegen soup and Qianghuoshengshi soup combined with acupuncture treatment cured 44 patients, 8 patients, and 2 patients; the total efficiency was 96.30% [13]
	Qing Bi Tang	Puerariae Lobatae Radix, Ephedrae Herba, Platycodonis Radix, Magnoliae Flos, Chuanxiong Rhizoma, Scutellariae Radix, Cinnamomi Ramulus, Paeoniae Radix Alba, Glycyrrhizae Radix et Rhizoma, Zingiberis Rhizoma Recens, and Jujubae Fructus	Wind-cold-dispersing medicine; clears heat and relieves stuffy nose	After FESS surgery, Qingbi decoction (Qing Bi Tang) was used prescribed to patients with chronic sinusitis to ameliorate symptoms and signs, reduce agglutination time, and promote the regression of diseases [14]
	Jin Fey Tsao saan	Ephedrae Herba, Paeoniae Radix Rubra, Glycyrrhizae Radix et Rhizoma, Zingiberis Rhizoma Recens, Jujubae Fructus, Schizonepetae Herba, Peucedani Radix, Inulae Flos, and Pinelliae Rhizoma	Wind-cold-dispersing medicine; directs qi downward to resolve phlegm	The obvious effect of using Jinfeicao powder is cough alleviation after infection [21]Jinfeicao powder has significant effects on reliving cough and expelling phlegm [15]
	Jie Geng	The dried root of Platycodon grandiflorum (Jacq.) and A. DC. (Campanulaceae)	Diffuses in the lungs and resolves phlegm, soothes the throat, and expels pus	PLD effectively inhibits the expressions of nuclear factor-*κ*B (NF-*κ*B), caspase-3, and Bax in lung tissues, restores the expression of Bcl-2 in the lungs, and improves superoxide dismutase (SOD) activity in bronchoalveolar lavage fluid (BALF) [16]
	Kan Chiang	The dried rhizomes of Zingiber officinale (Wild.) Rosc. (Zingiberaceae)	Promotes sweating to release the exterior, warms the middle to check vomiting, and warms the lung to suppress cough	Ginger extract reduces the expression of IL-17 and IL-23 in experimental autoimmune encephalomyelitis (EAE) mice [17]
	Xin Gren	The dried mature seed of Prunus armeniaca L. var. ansu Masim., Prunus sibirica L., Prunus mandshurica (maxim.) koehne, or Prunus armeniaca L. (Rosaceae)	Downbear counterflow suppresses cough and calms panting, moistens the intestines to relax the bowels	Amygdalin is one of the active ingredients in Armeniacae Amarum semen with antirenal interstitial fibrosis as pharmacological effect [18]

## Data Availability

Data used in this study are available within the article.

## Abbreviations

GGT: Ge Gen Tang
CXCTS: Chuan Xiung Cha Tiao San
YQS: Yin Qiao San
XYS: Xin Yi San
CWCHT: Chiu Wei Chiang Huo Tang
XYQFT: Xin Yi Qing Fei Tang
MXGST: Ma Xsing Gan Shih Tang
JFBDS: Jing Fang Bai Du Sa
SJY: Sang Ju Yin
XSS: Xing Su San
JFTS: Jin Fey Tsao Saan
MHFZXXT: Ma Huang Fu Zi Xi Xin Tang
QBT: Qing Bi Tang
QHSST: Qiang Huo Sheng Shi Tang
TCM: Traditional Chinese Medicine
NHIRD: National health insurance research database.

## Supplementary Materials

Supplementary Materials Table S1: shows the combination and ratio of formula, which listed in the study.
