# Supplementary material for: A Real-World Study on Ge Gen Tang in Combination with Herbal Medicines for Relieving Common Cold-Associated Symptoms
Source: Evid Based Complement Alternat Med. 2022 Jul 22;2022:4790910. doi: 10.1155/2022/4790910 (PMC9337945; doi:10.1155/2022/4790910)
Supplement: Supplementary Materials — Table S1: shows the combination and ratio of formula, which listed in the study. [file 4790910.f1.docx]

**Table S1.** The combination and ratio of formula which listed in the study.

| **Formula** | **Combination and Ratio** |
| --- | --- |
| Chuan Xiung Cha Tiao San (川芎茶調散) | Each 10.5g contains the following dry herbs:  Radix Angelica dahurica 1.5g, Radix Glycyrrhizae 1.5g,  Rhizoma et Radix Notopterygii 1.5g, Herba Menthae 6.0g.  Herba Schizonepetae 3.0g, Rhizoma Chuanxiong 3.0g,  Radix Asari 1.0g, Radix Saposhnikoviae 1.1g, |
| Yin Qiao San (銀翹散) | Each 12g contains the following dry herbs:  Fructus Forsythiae 5.0g, Flos Lonicerae 5.0g,  Radix Platycodonis 3.0g, Herba Menthae 3.0g,  Herba Lophatheri 2.0g, Radix Glycyrrhizae 2.5g,  Herba Schizonepetae 2.0g, Rhizoma Phragmitis 2.0g  Semen Sojae Praeparatum 2.5g, Fructus Arctii 3.0g. |
| Xin Yi San (辛夷散) | Each 12g contains the following dry herbs:  Flos Magnoliae 2.5g, Radix Asari 2.5g,  Rhizoma et Radix Ligustic 2.5g, Radix Angelicae Dahuricae 2.5g,  Rhizoma Cimicifugae 2.5g, Rhizoma Chuanxiong 2.5g,  Caulis Akebiae 2.5g, Radix Saposhnikoviae 2.5g,  Radix Glycyrrhizae 2.5g, Folium Camelliae 4.5g. |
| Chiu Wei Chiang Huo Tang (九味羌活湯) | Each 9g contains the following dry herbs:  Rhizoma et Radix Notopterygii 3.0g,  Radix Saposhnikoviae 3.0g, Rhizoma Atractylodis 3.0g,  Rhizoma Zingiberis Recens 3.0g,  Rhizoma Chuanxiong 2.0g, Radix Asari 1.0g  Radix Angelicae Dahuricae 2.0g, Radix Rehmanniae 2.0g,  Radix Scutellariae 2.0g, Radix Glycyrrhizae 2.0g. |
| Ma Huang Fu Zi Xi Xin Tang(麻黃附子細辛湯) | Each 6g contains the following dry herbs:  Ephedrae Herba 8.0g,  Radix Aconiti Lateralis Praeparata 5.0g,  Radix Asari 8.0g. |
| Qiang Huo Sheng Shi Tang  (羌活勝濕湯) | Each 6g contains the following dry herbs:  Rhizoma et Radix Notopterygii 5.0g,  Radix Angelicae Pubescentis 5.0g,  Rhizoma Chuanxiong 2.5g, Rhizoma et Radix Ligustic 2.5g,  Radix Saposhnikoviae 2.5g, Radix Glycyrrhizae 2.5g. |
| Qing Bi Tang(清鼻湯) | Each 9g contains the following dry herbs:  Radix Puerariae 3.6g, Ephedrae Herba 1.8g,  Rhizoma Zingiberis Recens 0.9g,  Radix Paeoniae Alba 1.8g, Radix Glycyrrhizae 0.6g,  Fructus Jujubae 1.2g, Cinnamomi Cortex 1,5g,  Rhizoma Chuanxiong 2.5g, Radix et Rhizoma Rhei 0.9g,  Semen Coicis 3.0g, Radix Platycodonis 2.7g,  Gypsum Fibrosum 1.2g, Flos Magnoliae 2.5g. |
| Jin Fey Tsao Saan  (金沸草散) | Each 6g contains the following dry herbs:  Herba Schizonepetae 6.0g, Radix Peucedani 4.5g,  Ephedrae Herba 4.5g, Flos Inulae 4.5g,  Radix Glycyrrhizae 1.5g, Rhizoma Pinelliae 1.5g,  Radix Paeoniae Rubra 1.5g, Fructus Jujubae 1.0g  Rhizoma Zingiberis Recens 3.0g. |
